# High Temporal Resolution 3D Live-Cell Imaging of Budding Yeast Meiosis Defines Discontinuous Actin/Telomere-Mediated Chromosome Motion, Correlated Nuclear Envelope Deformation and Actin Filament Dynamics

**DOI:** 10.3389/fcell.2021.687132

**Published:** 2021-11-25

**Authors:** Tadasu Nozaki, Frederick Chang, Beth Weiner, Nancy Kleckner

**Affiliations:** Department of Molecular and Cellular Biology, Harvard University, Cambridge, MA, United States

**Keywords:** 3D time-lapse imaging, chromosome motion, FROS, telomere, actin, homolog pairing, meiosis

## Abstract

Chromosome movement is prominent at mid-meiotic prophase and is proposed to enhance the efficiency and/or stringency of homolog pairing and/or to help prevent or resolve topological entanglements. Here, we combine fluorescent repressor operator system (FROS) labeling with three-dimensional (3D) live-cell imaging at high spatio-temporal resolution to define the detailed kinetics of mid-meiotic prophase motion for a single telomere-proximal locus in budding yeast. Telomere motions can be grouped into three general categories: (i) pauses, in which the telomere “jiggles in place”; (ii) rapid, straight/curvilinear motion which reflects Myo2/actin-mediated transport of the monitored telomere; and (iii) slower directional motions, most of which likely reflect indirectly promoted motion of the monitored telomere in coordination with actin-mediated motion of an unmarked telomere. These and other findings highlight the importance of dynamic assembly/disassembly of telomere/LINC/actin ensembles and also suggest important roles for nuclear envelope deformations promoted by actin-mediated telomere/LINC movement. The presented low-SNR (signal-to-noise ratio) imaging methodology provides opportunities for future exploration of homolog pairing and related phenomena.

## Introduction

Meiosis is the specialized cell cycle program which produces gametes with half the ploidy of their progenitor cells, thereby compensating for genome doubling at mating. This outcome is accomplished by occurrence of a single round of DNA replication followed by two rounds of chromosome segregation, meiosis I (MI) and meiosis II (MII). Homologous chromosomes (“homologs”) segregate to opposite poles at meiosis I. Sister chromatids then separate at meiosis II. Crossovers, the products of programmed homologous recombination, in combination with sister arm cohesion, allow the generation of tension between homologous chromosomes at metaphase of MI. This, in turn, ensures accurate distribution of homologs to opposite poles (Zickler and Kleckner, 1999, 2015).

Homologs achieve the configuration necessary for MI by a complex program of interactions that occupy a prolonged prophase stage. A major requirement of this program is that the homologs come close together in space, becoming aligned side-by-side (Zickler and Kleckner, 2015). During this process, two general problems must be addressed: (1) How can homologs meet one another on a reasonable time scale? This outcome requires both recognition of homology, e.g., via local events of DNA recombination, and global juxtaposition of whole chromosomes along their lengths. (2) What mechanism ensures the topological regularity of the pairing process so as to avoid or eliminate interlocks, entanglements, or ectopic pairing or non-homologous pairing (Storlazzi et al., 2010)?

With respect to time scale, the challenges of meiotic homolog pairing are illustrated by comparison with protein/DNA interactions. Proteins must scan the DNA information in the chromosomes to find their cognate binding sites (e.g., as for repressor proteins and transcriptional factors). By comparison, homology searching by chromosomes seems more difficult, from several points of view. First, the speed of chromosome motion in the eukaryotic G1 nucleus, as defined by analysis of movements of individually tagged loci over time, appears to be conserved, with diffusion coefficients ranging from 10^–4^ to 10^–3^ μm^2^/s (Marshall et al., 1997; Heun et al., 2001; Vazquez et al., 2001; Chubb et al., 2002; Dion et al., 2012; Miné-Hattab and Rothstein, 2012). This movement is much slower than that of proteins that are freely and three-dimensionally diffusing in the nucleus to identify their targets (10^0^–10^2^ μm^2^/s; e.g., Mazza et al., 2012; Chen et al., 2014; Izeddin et al., 2014; Normanno et al., 2015). Second, for protein/DNA interactions, many copies of the involved protein are searching for the target in parallel whereas, for meiotic chromosome pairing, there is only one pair of homologous chromosomes. Thus, the possibilities for parallel searches are more limited (discussion in Storlazzi et al., 2010).

In addition, both protein/DNA interactions and homolog pairing must solve the “speed-stability paradox” (Slutsky and Mirny, 2004). In brief, there is a tradeoff between the speed of searching and the stability required to ensure the desired cognate interaction. That is, for chromosomes, the same issue arises: homologs must be able to identify one another accurately without becoming trapped in nearly homologous interactions (Kleckner and Weiner, 1993; Bitran et al., 2017). In accord with these complexities, analysis of RecA-mediated homology recognition between a DNA locus and complementary oligonucleotide reveals that the rate of homologous pairing at the DNA level, which underlies meiotic homologous pairing (Zickler and Kleckner, 2015) is not rate-limited by the search for DNA homology (Yancey-Wrona and Camerini-Otero, 1995).

With respect to topological issues: entanglements/interlocks, and ectopic pairing between non-homologous chromosomes will impede the completion of homologous pairing and recombination and, eventually, clean segregation of homologous pairs at MI (Zickler, 2006; Koszul et al., 2008; Wanat et al., 2008). In fact, a modest number of topological interlockings are seen at the late leptotene and zygotene stages; however, they disappear during the pachytene stage, indicating that they can ultimately be resolved (Wettstein et al., 1984; Storlazzi et al., 2010).

These diverse challenges to homolog pairing seem severe. However, there are cellular mechanisms which help in overcoming them. One such mechanism is dynamic chromosome motion mediated by cytoskeletal forces exerted on telomeres through the nuclear envelope (described below). The current study focuses on the nature of these dynamic motions in budding yeast.

## Background

The first evidence of vigorous movements of chromosomes during meiotic prophase was found in rat spermatocytes (Parvinen and Söderström, 1976). By now, prophase movement has been described in a wide variety of organisms, albeit with some variations among different cases (e.g., Chikashige et al., 1994, 2006 in fission yeast; Scherthan et al., 2007; Conrad et al., 2008; Koszul et al., 2008 in budding yeast, Sheehan and Pawlowski, 2009 in maize, Christophorou et al., 2015 in *Drosophila*, Shibuya et al., 2014; Lee et al., 2015 in mouse, Sato et al., 2009; Baudrimont et al., 2010; Wynne et al., 2012; Link et al., 2018 in *C. elegans*). These movements have been proposed, variously, to promote homologous chromosome pairing, to reduce ectopic homologous pairing or pairing between near-homologous regions, and/or to resolve chromosome interlocks and entanglements (Koszul and Kleckner, 2009; Baudrimont et al., 2010; Lee et al., 2012; Wynne et al., 2012; Link and Jantsch, 2019). In accord with such possibilities, ectopic recombination and inappropriate telomere interactions are increased in budding yeast when motion is abrogated (Conrad et al., 2008; Lee et al., 2012) and in fission yeast when telomere clustering (a component of motion in that organism) is absent (Niwa et al., 2000; Davis and Smith, 2006). And in *C. elegans*, abrogation of motion results in inefficient pairing and entanglements (Penkner et al., 2007; Sato et al., 2009).

In most studied cases, vigorous meiotic prophase chromosome movement is generated by the linkage of chromosome ends to the cytoskeleton (reviewed in Koszul and Kleckner, 2009; Link and Jantsch, 2019). In such processes, mechanical forces of the cytoskeleton are transmitted to the telomeres through the nuclear membrane. Force transduction is provided by the Linker of Nucleoskeleton and Cytoskeleton (LINC) complexes, which is composed of inner nuclear membrane SUN domain proteins and outer nuclear membrane KASH domain proteins. Telomeres interact with SUN proteins, and KASH proteins bind to the cytoskeleton.

In budding yeast, dynamic prophase movement is dependent on the actin cytoskeleton (Scherthan et al., 2007; Conrad et al., 2008; Koszul et al., 2008). In this case, actin fibers are seen to “hug” the outer surface of the nuclear envelope in a curved path and telomeres are seen to move along those fibers, sometimes even after the fibers are no longer associated with the nucleus (Koszul et al., 2008; Supplementary Figures 1A,C; below). Visualization of whole pachytene chromosomes has also revealed that active movement of one telomere is accompanied by coordinate, spatially coordinated movements of other nearby chromosomes, often comprising half or more of the chromosome complement, whose motions are therefore “indirect” effects (Koszul et al., 2008; Supplementary Figure 1B). All of these motions are eliminated in the presence of Latrunculin B (“LatB”), a drug that prevents actin fiber polymerization by binding to actin monomers (Koszul et al., 2008).

Five molecules other than actin have also been implicated in actin-mediated motion (Fan et al., 2020; Lee et al., 2020). Ndj1 is a meiosis-specific protein that connects the telomere to the LINC complex via Mps3, a SUN domain protein that spans the nuclear membrane. Mps2 and Csm4 are related coiled coil proteins that interact with one another and mediate linkage of Mps3 to myosin Myo2 which, in turn, interacts with actin. Mutations that delete or alter any one of the involved proteins causes a severe reduction of rapid chromosome movement at mid-meiotic prophase and a delay in homolog pairing (Conrad et al., 2007, 2008; Scherthan et al., 2007; Kosaka et al., 2008; Wanat et al., 2008; Lee et al., 2012, 2020; Fan et al., 2020). Importantly, in mitotically dividing yeast cells, Myo2 mediates the transport of several different types of cargos along actin fibers (e.g., Beach et al., 2000; Schott et al., 2002). In the meiotic situation, this cargo comprises the entire nuclear envelope-embedded LINC complex as well as Ndj1 and its associated chromosome. Correspondingly, telomere movement is accompanied by nuclear envelope deformations nucleated at the telomere attachment site (Koszul et al., 2008; below).

In the present study, we studied telomere motion in budding yeast using FROS (fluorescent repressor operator system) labeling (Robinett et al., 1996; Straight et al., 1996; Marshall et al., 1997; Heun et al., 2001) plus a unique methodology for 3D live-cell imaging and spot detection at high spatio-temporal resolution and low signal-to-noise ratio (SNR), as developed in our laboratory (Chang, 2018). Trajectories of single labeled telomeres were defined in 3D at 500 ms intervals over total time spans of 6 min. This approach provides a previously unavailable description of the detailed dynamics of an individual telomere during meiotic mid-prophase. For comparison, we analogously examined telomere movements at premeiotic G1/G0 and in selected mutant situations (details in section “Materials and Methods” and below).

Concomitantly, the boundary of the nucleus, and thus by implication the nuclear envelope, was visualized using a general nuclear signal, with the position of the labeled telomere locus localized relative to that signal. Meiotic prophase actin filament dynamics were also visualized, thus allowing assessment of their contribution to net telomere movement. The presented findings provide new information regarding the nature of actin-mediated chromosome motions in this system.

## Materials and Methods

### Yeast Strains

*Saccharomyces cerevisiae* strains are *MATa/MATα* derivatives of wild-type SK1 as follows: *scp1::tetO array::LEU2*/*SCP1, leu2::URA3p-TetR-mEGFP::LEU2/leu2::hisG* (TNY570); *scp1::tetO array::LEU2*/*SCP1, leu2::URA3p-TetR-mEGFP::LEU2/leu2::hisG*, *ndj1*Δ*/ndj1*Δ (TNY669), *ABP140-4xGFP::KanMX*/*ABP140-4xGFP::KanMX* (YKK389), *SPC42-YFP::URA*/*SPC42* (TNY866). TetO array system is based on Kim et al. (2010).

### Meiotic Time Course

All operations were performed at 30°C. Strains maintained in glycerol stock at −80°C are patched onto YEPG plates (3% w/v glycerol, 2% w/v bactopeptone, 1% w/v yeast extract, 2% w/v bactoagar) overnight. Cells were struck out to single colonies on YEPD plates (2% w/v bactopeptone, 1% w/v yeast extract, 2% w/v glucose, 2% w/v bactoagar) and grown for 2 days. A single colony is transferred to 4 ml YEPD liquid medium (2% w/v bactopeptone, 1% w/v yeast extract, 2% w/v glucose) and grown overnight. A 1/100 dilution of the culture was made with YEPA medium (1% w/v potassium acetate, 2% w/v bactopeptone, 1% w/v yeast extract, 2 drops per liter antifoam) and grown for 13.5 h. Meiosis was initiated by transfer of cells to 1% sporulation medium (SPM) (1% w/v potassium acetate, 0.02% w/v raffinose, 2 drops per liter antifoam). We note that this series of preparation conditions is specifically defined in such a way that, at the time of transfer to the SPM, cells have completed ongoing mitotic cell cycles and are becoming larger without initiating a new mitotic cycle and thus are present primarily as large unbudded cells. Upon transfer to SPM, this population initiates meiosis synchronously and efficiently (e.g., Kim et al., 2010).

### Chemical Treatment of Meiotic Cells

Latrunculin B (LatB) was from Santa Cruz Biotechnology Inc. and was dissolved in dimethyl-sulfoxide (DMSO). LatB was added to a final concentration of 30 μM at 2 h after initiation of meiosis by transfer to SPM and imaged after an additional 2 h (*t* = 4 h of meiosis).

### Live-Cell Imaging

Cell samples from an experimental culture were vortexed at full speed and 1 μl quickly spread onto a glass base dish (MatTek) coated with Concanavalin A (ConA). A premade agarose pad (1%) was placed on top of the cell drop and excess media was absorbed by a piece of Kimwipe. Cells were observed at 30°C using a Ti microscope (Nikon) equipped with GFP filters (Semrock), a sCMOS camera (Hamamatsu Photonics), and a piezo device (Physik Instrumente) for acquiring Z stacks. Cells were exposed to the LED light (Lumencor) through an objective lens (60× PlanApo, NA 1.40; Nikon). The microscopy system was controlled, and images were acquired, through μ-Manager software and MATLAB.

For short time scaled imaging, movies of 15 z-stack steps with 389-nm step size and 720 sequential frames were acquired using μ-Manager software with 25 ms exposure time and 8.3 ms camera acquisition time, totaling 500 ms for one set of z-stack images. For long timescale imaging, movies of 13 z-stack steps with 389-nm step size were acquired using μ-Manager software with 10 ms exposure time and 16.6 ms camera acquisition time. Z-stacks were acquired at 1 min interval for 10 h, giving a total of 601 z-stack images.

Images were denoised quantitatively by a home-made program that accurately defines the presence and positions of fluorescent spots at very low signal-to-noise ratios (Chang, 2018). This algorithm dramatically extends the ability to capture many images over long periods of time, as illustrated in this work. Spots were detected and tracked by ImageJ Fiji plug-in TrackMate (Schindelin et al., 2012; Tinevez et al., 2017). The distribution of spot displacements per time interval and the mean square displacement (MSD) of the fluorescent signals were calculated in 3D based on this TrackMate data. The MSD was calculated by the following formula. MSD = <(*X*_*t*_ − *X*_*t* + Δ*t*_)^2^>. *X*_*t*_ was the three-dimensional position of a fluorescent spot at time t (Seeber et al., 2013). The multi-component Gaussian mixture model is fitted to the step size data of the spot motion using mixtools in R packages (Benaglia et al., 2009). To eliminate the influence of large step size (Group 1, see below) to the 5 s interval analysis, the ± 10 frames (5 s) from the frame that shows the large step in 500 ms were eliminated. We note that, due to specific features of our imaging system and spot detection algorithm, the spatial precision of spot detection in our system is high and that there is no detectable photobleaching or phototoxicity, as discussed in the text and also in Supplementary Notes.

### Fixed-Cell Imaging

For fixed cell analysis, cells were transferred to 4% paraformaldehyde at 4 h after initiation of meiosis as described above. Cells were then spun down and the cell pellet was resuspended in 0.1 M potassium phosphate for imaging. Other procedures are the same as for live-cell imaging.

## Results

**FIGURE 1:**
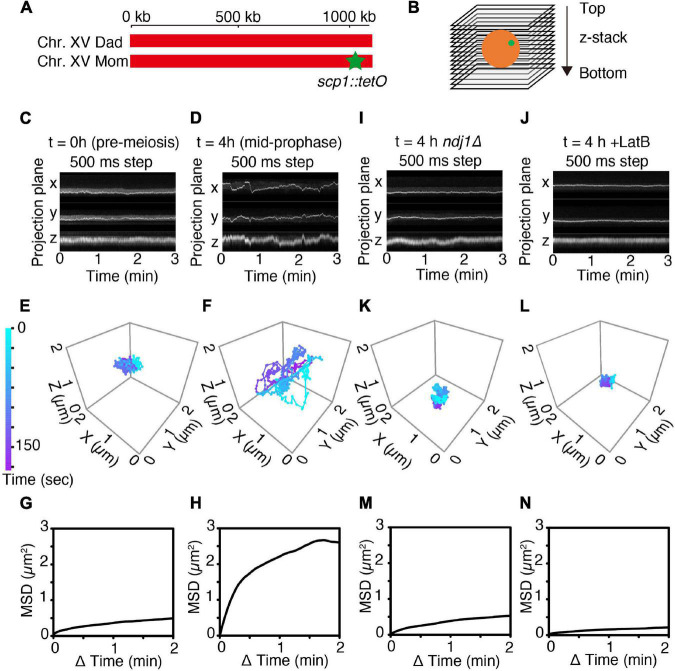
4D imaging of telomere trajectories. **(A)** Chromosome labeling of the *SCP1* locus with TetR-mEGFP. The end of only one chromosome (chromosome XV) in a diploid cell is labeled. **(B)** The scheme of 3D live-cell imaging in budding yeast. **(C,D)** Kymographs of representative cells tracked in 3D at *t* = 0 h (**C**; pre-meiotic G1/G0) and at *t* = 4 h (**D**; mid-meiotic prophase) cultured in SPM. 3D images are captured once per 500 ms. **(E,F)** Trajectories of the fluorescent spots shown in **(C,D)** as tracked in 3D at pre-meiotic G1/G0 (*t* = 0 h) **(E)** and at meiotic prophase (*t* = 4 h) **(F)**. **(G,H)** MSD curves of the tagged *SCP1* locus in cells at pre-meiotic G1/G0 (G; *n* = 10 cells) and at meiotic prophase (H; *n* = 17 cells). **(I–N)** Kymographs, representative trajectories, MSD curves for *ndj1Δ* mutant cell(s) (**I,K,M**; *n* = 10 cell) and LatB-treated cell(s) (**J,L,N**; *n* = 10 cells) exactly as in the corresponding **(C,E,G)**.

To measure telomere-led chromosome movement, we constructed a diploid strain in which one chromosome carries a sub-telomeric fluorescent tag. The *SCP1* locus is located 64 kb from the right arm telomere of chromosome XV. An array of binding sites (*tetO*) for the tetracycline resistance repressor was inserted into one chromosome of a strain also carrying that repressor fused to mEGFP (TetR-mEGFP), giving a fluorescent focus corresponding to *scp1::tetO*/TetR-mEGFP (Figure 1A). Motion of the tagged locus was defined by imaging of the corresponding fluorescent spot in 3D (Figure 1B) at 500 ms intervals, typically for a total of 6 min (section “Materials and Methods”). We note that the approaches we use allow us to accurately define the position of a fluorescent spot, to ∼0.040 μm in the X and Y dimensions and ∼0.075 μm in the Z dimension. The accuracy of our imaging system and spot detection were estimated by imaging of the fixed cells and calculating the standard deviation of the detected spot position (see “Materials and Methods,” Supplementary Figures 2A,B and Supplementary Note; Nozaki et al., 2013; Miné-Hattab et al., 2017). These values, which we consider to be the upper bound of localization accuracy, are lower than the 500 ms step sizes observed in any of data reported below, implying that the movements described below faithfully reflect actual telomere movement.

### Motion Is Dynamic at Mid-Prophase as Compared to Premeiotic G1/G0

Telomere motion was examined analogously in premeiotic G1/G0 cells (defined as large unbudded cells observed immediately upon transfer to sporulation medium; “Materials and Methods,” i.e., *t* = 0 h, Figures 1C,E,G) and in meiotic prophase cells observed at *t* = 4 h after initiation of meiosis, the time at which dramatic chromosome movements are known to occur (Koszul et al., 2008; see Supplementary Figure 4 and Figures 1D,F,H). Parallel control experiments confirm that cells are at late zygotene or early pachytene at this time, as judged by Zip1-GFP patterns (T.N. unpublished).

In accord with previous observations, telomere motion is limited at pre-meiotic G1/G0 (*t* = 0 h) and dramatic at meiotic mid-prophase (*t* = 4 h). This difference is qualitatively apparent in representative kymographs which show the position of the spot as projected onto the X-, Y-, or Z-plane over a 3 min time period (Figure 1C vs. Figure 1D) and in corresponding 3D trajectories (Figure 1E vs. Figure 1F). At G1/G0, the telomere locus never moves far from its starting point in 3 min (Figure 1E) while at mid-prophase, it traverses distances comparable to the diameter of the nucleus (∼2 μm) (Figure 1F).

The difference between the two situations can be described quantitatively. Plots of mean square displacement (MSD) over time are widely used with single-particle tracking to quantify chromatin and chromosome dynamics (Heun et al., 2001; Sage et al., 2005; Miné-Hattab and Rothstein, 2012; Hajjoul et al., 2013; Nozaki et al., 2017). For normal diffusion, the MSD of a tracked “spot” varies linearly with time. In contrast, at both G1/G0 and mid-prophase, the marked telomere exhibits a rising curve which reaches a plateau. This behavior, referred to as “anomalous diffusion,” implies that the movement of the tracked particle is somehow constrained, with the distance reached at the plateau defined as the “radius of constraint” (Rc) (Neumann et al., 2012). Confinement is severe at G1/G0 (Rc = 0.64 μm; Figure 1G, *n* = 10 cells). Interestingly, this pattern is very similar to that observed by analogous MSD analysis in yeast mitotic interphase cells (Sage et al., 2005). Confinement is much less severe at mid-prophase, with Rc = 1.46 μm, roughly comparable to the diameter of the nucleus (Figure 1H, *n* = 17 cells), in accord with the fact that telomeres are actively moved around the nuclear periphery during this period (Koszul et al., 2008). The mid-prophase MSD curve also rises more sharply than the G1/G0 MSD curve. This difference implies that the telomere locus tends to move farther in a given period of time in mid-prophase vs. G1/G0, in accord with the presence and absence of active actin/Myo2-mediated movements in the two situations, respectively.

### Meiotic Prophase Telomere Motions Are Driven by Actin/Telomere Linkage

Mid-prophase telomere motions are substantially reduced by deletion of meiotic telomere protein Ndj1 (which connects telomeres to the LINC complex; Background) (*ndj1Δ*; Figures 1I,K,M). These movements are also dramatically reduced by treatment with LatB, which blocks actin polymerization (Figures 1J,L,N). Thus, as expected, the dynamic prophase motions of telomere-located *scp1::tetO*-TetR observed at mid-prophase are derived from the force generated by the linkage of the telomere to cytoskeletal actin fiber(s) via LINC complexes.

Interestingly, the overall behavior of telomeres in *ndj1Δ* is similar to that observed at G1/G0 (compare Figures 1I,K with Figures 1C,E; further discussion below). Also, the effect of LatB is more severe than that of *ndj1Δ*, perhaps because telomeres are now locked onto immobile LINC complexes rather than being free of such complexes.

### Analysis of Mid-Prophase Telomere Movement Reveals Three General Categories of Motion

#### Background

All previous studies identify occasional (sporadic) extremely rapid, straight/curvilinear telomere movements which occur over very short time period and are attributable to direct transport of a monitored telomere along an actin filament (e.g., Conrad et al., 2008; Koszul et al., 2008; Lee et al., 2012, 2020; Discussion). Additional insight is provided by analysis of whole pachytene chromosomes in which the movements of many/most chromosomes were directly visualized simultaneously over time (e.g., Koszul et al., 2008; Supplementary Figure 1B). Such images reveal three types of telomere motion (Supplementary Figure 1B). First, one telomere may move rapidly in a highly directed fashion over a very short time period (∼3 s) corresponding to transport of this “lead” telomere along an actin fiber. Second, this movement is accompanied by coordinate movement of a multiple chromosome group in the same direction as the “lead” telomere, with accompanying dramatic changes in overall nucleus shape. These coordinated motions comprise a second type of actin-dependent telomere motion which, however, is promoted indirectly by directly promoted motion of the “lead” telomere. Third, even despite the above progressions, many chromosomes (and thus their telomeres) do not move. Importantly, for given monitored telomere as in the present study, indirectly promoted movements can be provoked by any of the other (unmonitored) 31 telomeres (16 chromosomes x 2 telomeres) in the nucleus and thus will be much more frequent than sporadic direct transport movement. The same is true for pauses which, at any given moment, pertain to many telomeres (Supplementary Figure 1B) and thus will occupy a substantial lifetime of any one monitored telomere. For convenience, we refer to the telomere motions in these three conditions as “directly promoted,” “indirectly promoted,” and “pause.”

#### Two Approaches to Analysis of Mid-Prophase Telomere Motion

We were interested to understand whether telomere motions as detected by spot tracking could be seen to comprise distinct categories, e.g., as revealed by whole chromosome analysis (above).

- In one approach we analyzed the distributions of distances traveled by the monitored telomere over intervals of 500 ms and 5 s (11 image frames). 500 ms step sizes, being shorter, give a clearer impression of the intrinsic rate of telomere movement. 5 s step sizes, in contrast, reflect not only the intrinsic rate of movement but the extent to which that movement is or is not “directed.” Without directionality, sequential steps are randomly oriented and the telomere remains close(r) to its starting point after 5 s. If motion is in any manner directional, sequential steps tend to occur in the same direction and the telomere tends to move farther from its starting point in 5 s, in relation to the straightness of the trajectories.

- In a second approach we inspected movies of 3D projections visually to determine whether any specific trajectory patterns might reproducibly appear out of the generally chaotic motions, thereby identifying different distinct “motion phenotypes.”

The former approach has the advantage of considering the totality of the data. The latter approach has the advantage of including spatio-temporal information, i.e., the added dimensions of multi-step trajectory and duration, neither of which appear as inputs into step-size analysis. In addition, this approach allows the biological situation to highlight particularly clear or prominent types of movement.

Both approaches reveal the same underlying reality: the majority of telomere movements and trajectories fall into three broad categories which correspond to one another and to the three general categories expected on the basis of whole chromosome analysis as described above.

#### Categories of Telomere Movements as Revealed by Step Size Analysis

**FIGURE 2:**
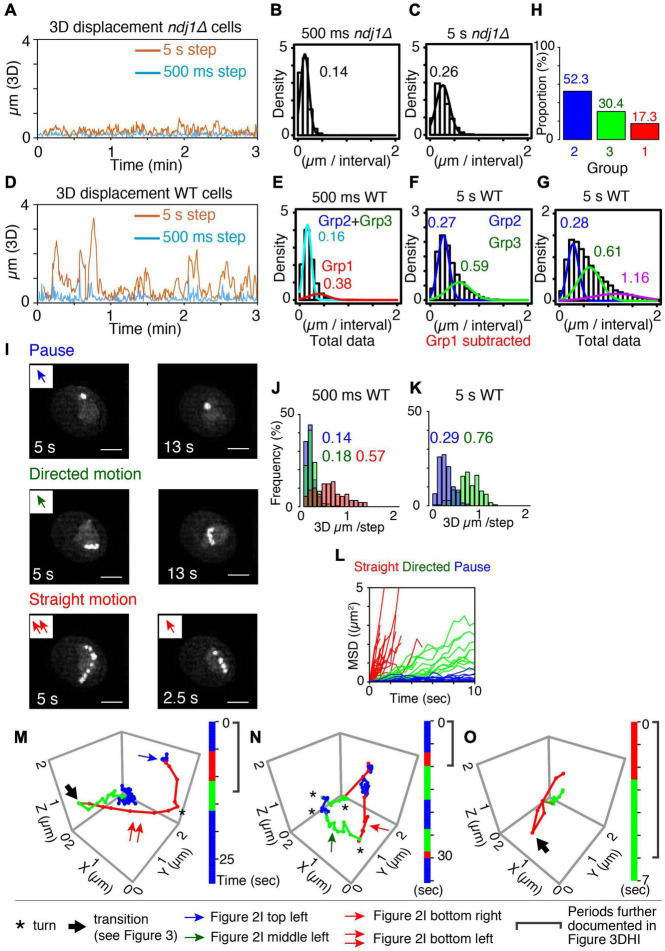
Categorization of meiotic prophase telomere motion. **(A)** Representative 3D displacement pattern for 3 min with 500 ms step (turquoise) and 5 s step (orange) in *ndj1Δ* cells at *t* = 4 h. **(B,C)** Distribution of distances traveled (step size) over 500 ms (**B**, mean = 0.14 μm) and 5 s intervals (**C**, mean = 0.26 μm) plotted with Gaussian model in *ndj1Δ* cells at *t* = 4 h (*n* = 10 cells). **(D)** Representative 3D displacement pattern for 3 min with 500 ms step (turquoise) and 5 s step (orange) in WT cells at *t* = 4 h (mid-prophase). **(E–G)** Distribution of distances traveled over 500 ms [**E**, mean = 0.16 μm (cyan, Group2+Group3) and 0.38 μm (red, Group 1)], 5 s intervals [**F**, mean = 0.27 μm (blue, Group 2) and 0.59 μm, (green. Group 3)], and 5 s intervals [**G**, mean = 0.28 μm (blue, Group 2), mean = 0.61 μm (green, Group 3), and mean = 1.16 μm (purple)] plotted with the two or three component Gaussian mixture model in wild type cells at = 4 h (*n* = 17 cells). In **(F)**, the trajectories that include >0.38 μm/500 ms (Group 1) were eliminated. In **(G)**, the trajectories classified in purple include at least one step from Group 1 eliminated in **(F)**. **(H)** The proportion of the Group 1, 2, and 3 estimated from **(E,F)**. **(I)** The projection images of the totality of positions observed in the XY plane over the duration of the trajectory for these three types, “pause” (top), “directed motion” (middle), and “straight motion” (bottom) are illustrated. The scale bar is 2 μm. Bottom left includes two continuous straight motion with the abrupt turn. **(J–L)** Comparison between distributions of distances traveled (step sizes) for the three types of mid-prophase motion over 500 ms **(J)** and 5 s intervals **(K)**. Comparison between MSD curves for the three types of prophase motion **(L)** (*n* = 17 trajectories in pause, *n* = 18 trajectories in directed motion, *n* = 28 trajectories in straight motion from 7 cells). **(M–O)** Three representative series of trajectories for a tracked telomere locus at meiotic prophase (*t* = 4 h). “Pause” = jiggling spot (blue). “Straight motion” = rapid motion in a straight/slightly curved trajectory (red). “Directed motion” = directional movement with a “zig-zag” path rather than a straight path (green). Vertical bars indicate the duration of each trajectory segment. Total imaging time represented was ∼30, 35, and 7 s, respectively. A single asterisk (*) indicates a sharp change in direction. A large arrow indicates transition of the motion (recoil motion) (see text). The blue arrow in **(M)** indicates the trajectory in **(I)** left top. The red arrow in **(M,N)** indicates the trajectory in **(I)** left bottom and right bottom, respectively. The green arrow in **(N)** indicates the trajectory in **(I)** left middle.

We compared mid-prophase telomere motions in wild type meiosis with those in *ndj1Δ*, where telomeres are not associated with LINC complexes and are thus not subject to either direct or indirect effects of actin/Myo2-mediated movement. In *ndj1Δ*, the array of distances of movement over time shows a low baseline of 0.1 μm and 0.2 μm at 500 ms and 5 s intervals, respectively (Figure 2A and Supplementary Figure 3A). Motions at G1/G0, where telomere/LINC associations are also absent, are very similar (Supplementary Figures 3B,E). In striking contrast, in wild type meiotic mid-prophase, step sizes fluctuate dramatically over time, from 0 to 1 μm for 500 ms intervals and 0–3 μm for 5 s intervals (Figure 2D). The most dramatic motions are comparable to those observed previously for individual tracked telomeres (Lee et al., 2012, 2020); the smallest motions are comparable to those observed in *ndj1Δ*; and many distances fall between these two extremes, at both step sizes.

For *ndj1Δ*, at both 500 ms and 5 s intervals, total step sizes exhibit the single-peaked curve, well-fitted by a Gaussian model, with average distances traveled of 0.14 μm/500 ms and 0.26 μm/5 s, respectively (Figures 2B,C). Motions at G1/G0 are very similar (0.19 μm/500 ms and 0.32 μm/5 s, respectively; Supplementary Figures 3C,D). In both cases, the marked telomere is moving relatively slowly and without directionality, i.e., is essentially “jiggling in place” (e.g., Figures 1E,K). These patterns provide a baseline description of mid-prophase telomere movement in the absence of telomere-LINC association. Similar results were observed in LatB-treated cells but with even a bit smaller step sizes than wild type cells (0.13 μm/500 ms and 0.20 μm/5 s, respectively, Supplementary Figures 3F–I).

For wild type telomere motion, the distribution of 500 ms step sizes exhibits a prominent peak with a mean value of 0.16 μm, plus a discernible tail of much greater movement with a mean value of 0.38 μm. This pattern is well-described by a corresponding two-component Gaussian mixture model (Figure 2E). The component corresponding to the minor tail (denoted “Group 1”) exhibits a very large step size, corresponding to those seen previously and attributed to direct actin-mediated transport (above; Conrad et al., 2008; Koszul et al., 2008; Lee et al., 2012, 2020). In contrast, the major subcomponent distribution exhibits a relatively small average step size which is very similar to that observed for *ndj1Δ*.

To further dissect the nature of this latter majority group of motions, we eliminated the subset of very large steps (>0.38 μm/500 ms) from the total data set (see “Materials and Methods”) and analyzed the distribution of 5 s step sizes for the remaining (vast majority of) motions. A broad distribution emerges which is well-fitted by a two-component Gaussian mixture model. One component has a mean step size (0.27 μm) which again corresponds to the 5 s step size of *ndj1Δ* (0.26 μm) while the other has a larger mean step size (0.59 μm) (“Group 2” and “Group 3,” respectively; Figure 2F). Thus, over longer (5 s) intervals, the telomere often exhibits *ndj1Δ*-like behavior but also frequently travels significantly farther than in the *ndj1Δ* case (Figures 2C,F). Since all of these motions had similar 500 ms step sizes (above), the difference between the two categories can be attributed to different tendencies for straightness of motion. In the Group 2 component, telomeres are “jiggling in place” as in *ndj1Δ* and in the Group 3 component, telomeres are exhibiting significantly more directed motion. Importantly, the fact that the second component is absent in *ndj1Δ* further implies that it reflects actin-dependent movement.

When taken together, these analyses define three categories of motion (Groups 1, 2, and 3). In accord with these interpretations, the distribution of 5 s step sizes for the totality of all data, including very fast 500 ms movements, can be fitted by a corresponding three-component Gaussian mixture model which now includes a set of step sizes that are dramatically larger than those of Groups 2 and 3 (Figure 2G). The data further show that rapid movements (Group 1) are rare, comprising ∼17% of total imaging time while the other two categories are both quite frequent, comprising 52% and 30% of total imaging time, respectively (Figure 2H).

The properties of these three groups directly match those predicted from whole chromosome analysis. Group 1 motions, which are very rapid, highly directional and rare, correspond to directly promoted Myo2/actin-mediated transport of the monitored telomere. Group 2 motions, which are abundant, slow, and non-directional, analogous to *ndj1Δ* “jiggling in place,” correspond to motions of telomeres which are paused. Group 3 motions are abundant and directional, but only modestly so as compared to directly promoted motions (Group 1). These are the features predicted for indirectly promoted effects driven by directly mediated motion of an unmarked telomere. Notably, also, telomeres spend the vast majority of the time in Group 2 and Group 3 (∼80%), which have the same or very similar 500 ms step sizes. This correspondence is directly explained by the inferred assignments. The only difference between the two categories is whether a telomere is not, or is, being subjected to indirect forces from movement of an unmonitored telomere. Thus, their intrinsic motions are expected to be the same, but with a modest directional bias for Group 3.

#### Categories of Telomere Movements as Revealed by “Motion Phenotypes”

Visual inspection of 3D movies of telomere motion in wild type mid-prophase revealed three distinct patterns of motion that were clearly detectable above the background of other fluctuations. Two of these “motion phenotypes” are visually obvious because they exhibit specific diagnostic patterns that persist over long periods.

##### Pauses

A telomere is, to the eye, essentially immobile over 5–15 s (10–30 frames) (Figure 2I top panels and Figures 2M,N blue). This is the same phenotype observed for *ndj1Δ* where, however, it persists for the entire duration of the movie (Figure 1K and Supplementary Figure 3A). This phenotype corresponds to that defined as pausing of telomere motion observed in whole chromosome analysis. Correspondingly, the 500 ms and 5 s step sizes of these visually identified pause periods correspond closely to those observed for *ndj1Δ* and for wild type Group 2 in step-size analysis of total data sets (Figures 2J,K blue vs. Figures 2B,C,E–G).

##### Directed Motion

A telomere moves relatively slowly in a straight or curvilinear path that again lasts for 5–15 s (Figure 2I middle panels and Figures 2M–O green). This phenotype corresponds to the motions of telomeres seen during indirectly mediated motion in whole chromosome analysis (Supplementary Figure 1B). Correspondingly, the 500 ms and 5 s step sizes of these trajectories correspond closely to those observed for wild type Group 3 in step-size analysis of total data sets (Figures 2J,K green vs. Figures 2B,C,E–G).

##### Straight Motion

A third type of motion is obvious to the eye because it involves a very distinctive type of movement: very fast, very straight/curvilinear motion through a substantial distance in a single multi-frame a trajectory that lasts only ∼3 s or less (Figure 2I bottom panels). These spatio-temporal properties match the defining properties of actin-mediated movement in all types of studies (above). Correspondingly, the 500 ms step sizes for straight motions correspond to those defined above as wild type Group 1 (Figure 2J red and Figure 2E). In fact, on a per-frame basis, the trajectories that fall into this category mostly comprise exactly the same steps as those which comprise the wild type Group 1 component (data not shown). We also note that, while most of these trajectories are too short to define 5 s step sizes, their contributions to the distribution of 5 s step sizes in the total data set are nonetheless manifested in a third component to the Gaussian mixture model (Figure 2G, purple). Moreover, we can infer that the step size of that third component is a substantial underestimate because any 5 s step size that includes directly mediated motion will also include other type(s) of movement.

The MSD relationships for these three phenotypic classes also correspond to the expected descriptions, with highly constrained, modestly constrained, and completely unconstrained motion, respectively (Figure 2L).

#### Synthesis

Two independent approaches, each with unique strengths and weaknesses, converge on a single conclusion: telomere movements as defined by spot-tracking at high spatio-temporal resolution can, to the first approximation, be explained by three distinct modes of telomere movement which also correspond to those manifested in whole chromosome images obtained in an earlier study (Koszul et al., 2008): directly promoted Myo2/actin-mediated movement; pauses in which the telomere “jiggles in place”; and periods when the monitored telomere is being moved due to indirect effects resulting from directly promoted movement of an unmonitored telomere.

### Whole Nucleus Motions Occur but Are Rare and Do Not Explain Most Telomere Movements

It is well known that cytoskeletal forces can also provoke whole nucleus movements during meiotic prophase (e.g., in budding yeast, Conrad et al., 2008). To evaluate the potential contribution of such movements to telomere locus dynamics, we tracked the SPB component SPC42, tagged with YFP (Koszul et al., 2008). Since the SPB is embedded in the nuclear envelope throughout prophase (Koszul et al., 2008), its motions are often used to define whole nucleus movements within the cell in meiosis (Conrad et al., 2008) and in mitotic cells (Heun et al., 2001). This analysis suggests that whole-nucleus movements do not contribute significantly to either “directed” or “straight” motions; however, they likely account for some of the motions seen as “pauses.” This conclusion is validated by three findings:

(i) 500 ms step sizes of SPB are small (0.11 μm/500 ms), even slightly smaller on average than for pauses (0.14 μm/500 ms; Supplementary Figure 2D left vs. Figure 2J blue).
(ii) 5 s step sizes of SPB (0.31 μm/5 s) are similar on average to those of pauses (0.29 μm/500 ms). Interestingly, this distribution is skewed to lower values with a tail of slightly higher values, in accord with occasional more extensive movement (Supplementary Figure 2D right vs. Figure 2K blue).
(iii) MSD curves for SPB trajectories are usually flat, similar to those of pauses (Supplementary Figure 2E compared with Figure 2L blue), although there are rare exceptions (Supplementary Figure 2E, asterisks).

Finally, we note that additional global whole nucleus movements may tend to occur over longer time scales than the 6 min duration of imaging used in the present analysis.

### Telomere-Nuclear Envelope Association Is Always Present

**FIGURE 3:**
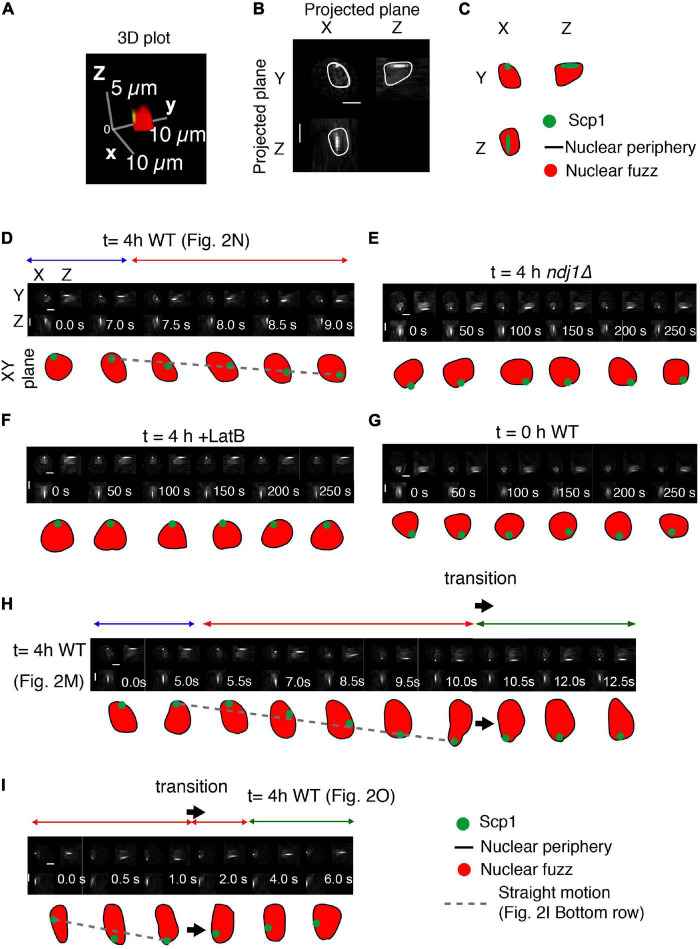
Interplay between telomeres and nuclear envelope. **(A–C)** Analysis of a representative nucleus showing the definition of the nucleus, based on the nuclear fuzz (red) that is derived from the unbound TetR-mEGFP in the nucleus, and thus the nuclear periphery (NP; black line) plus the position of the spot corresponding to *scp1::tetO*-TetR-mEGFP. **(A)** 3D representation. **(B)** Projections onto the XY, XZ, and YZ planes. **(C)** Interpretation. **(D–I)** Projected images of the nuclear region and tagged *SCP1* locus with the nature of spot motion (above; horizontal arrows). Sequences in **(D,H,I)** are from the trajectories shown in Figures 2M–O, respectively. Scale bar is 2 μm.

Previous time-lapse data of prophase movements indicated that telomeres remain associated with the nuclear envelope during periods of movement and during pauses (Koszul et al., 2008). We now show that this association is maintained throughout the entire progression of complex motions described above. Here, the 3D outline of the nucleus can be defined by the “fuzz” of fluorescence from TetR-mEGFP that is nuclear localized but not specifically bound to the chromosomes that reflects its overall nuclear localization (Figures 3A–C). Three-dimensional reconstructions show that during the mid-prophase dynamic motion period (*t* = 4 h), the tagged telomere locus is attached to (or closely associated with) the nuclear periphery at all times, irrespective of whether it is exhibiting straight, directed or paused motion (as defined by visual inspection; Figures 3D,H,I).

Interestingly, the telomere is also associated with the nuclear periphery in the *ndj1Δ* mutant (Figure 3E). The same is true during premeiotic G1/G0 (Figure 3G) and is also known to be the case for telomeres in mitotic cells (Sage et al., 2005). In all three cases, such association is independent of the meiosis-specific LINC complex, which is absent in *ndj1Δ* (Conrad et al., 2008) and unformed during pre-meiosis and in mitotic cells. This condition of LINC-independent nuclear envelope association can thus be inferred to underlie the “jiggling in place” movements observed in all three situations. By extension, telomeres might also be in this state during paused and indirectly promoted movements in wild type meiosis (Discussion). Telomeres also remain associated with the nuclear surface in LatB treated cells (Figure 3F). Whether telomeres remain associated with LINC complexes in this condition or not remains to be determined. It is possible that absence of an actin filament triggers disassembly of LINC complexes to give *ndj1Δ-*like associations with the nuclear periphery also in this situation (Discussion).

### Nuclear Envelope Deformations and Telomere Recoil

We sometimes observe cases in which a period of straight telomere movement is immediately followed by a rapid reverse motion (Figures 2O, 3I, large arrow). These “transition” effects are reminiscent of the previously described “recoil” of FROS-tagged loci (Conrad et al., 2008). We further find that a transition involving such reverse motion is accompanied by change in the shape of the nuclear surface (Figures 2M, 3H, large arrow). The nucleus initially elongates concomitantly with, and in the same direction as, an outward-directed straight telomere movement; then, beginning at the time of telomere recoil, the nucleus returns to its normal, roughly spherical, shape. We suggest that a complex between a telomere/LINC/nuclear envelope becomes attached to a nucleus-hugging actin fiber and then follows that fiber away from the nucleus, dragging the associated nuclear envelope region with it. If the telomere complex is then released from the actin filament, the result would be recoil of the telomere and restoration of nuclear shape. A general association of nuclear shape changes at points of telomere attachment (Koszul et al., 2008) and during rapid prophase chromosome movements (Conrad et al., 2008) have been described previously. Among these are cases of long actin-mediated nuclear protrusions with (or without) an associated orphan chromosome (Koszul et al., 2008). In such cases, pulling on the nuclear envelope results in separation of the nuclear membrane from constraining features to give a long “membrane tube.” We suggest that the global telomere-linked nuclear deformations identified in the current analysis could be a major factor in limiting the duration of direct Myo2/actin-promoted telomere motion and an important contributor to indirectly promoted telomere movements (Discussion).

### During Meiotic Prophase, Actin Fibers Move Dynamically Within the Nucleus While Concomitantly Undergoing Treadmilling

Movement of an actin-linked telomere through space will be the net result of the combined effects of Myo2-mediated tracking along an actin fiber and motion of the actin fiber itself. To further elucidate the contributions of the latter effect, we visualized ABP140-GFP signals in mid-prophase of meiosis.

Visualization of actin fibers in mitotic cells of budding yeast has defined two types of movement: dynamic movements of filaments through 3D space and treadmilling, in which subunits are added to one end and lost from the other (Yang and Pon, 2002). The two types of dynamics can be defined and distinguished by analysis of local strongly staining regions which provide fiduciary marks (Yang and Pon, 2002). Movement of the actin fiber through space (irrespective of treadmilling) is indicated by movement of a fiduciary mark. Treadmilling is implied by changes in the lengths of the fiber segments on either side of the fiduciary mark, with growth at the (+) end and shortening at the (−) end. In the present study, by applying analogous methods, we could see both types of processes occurring during meiotic prophase.

**FIGURE 4:**
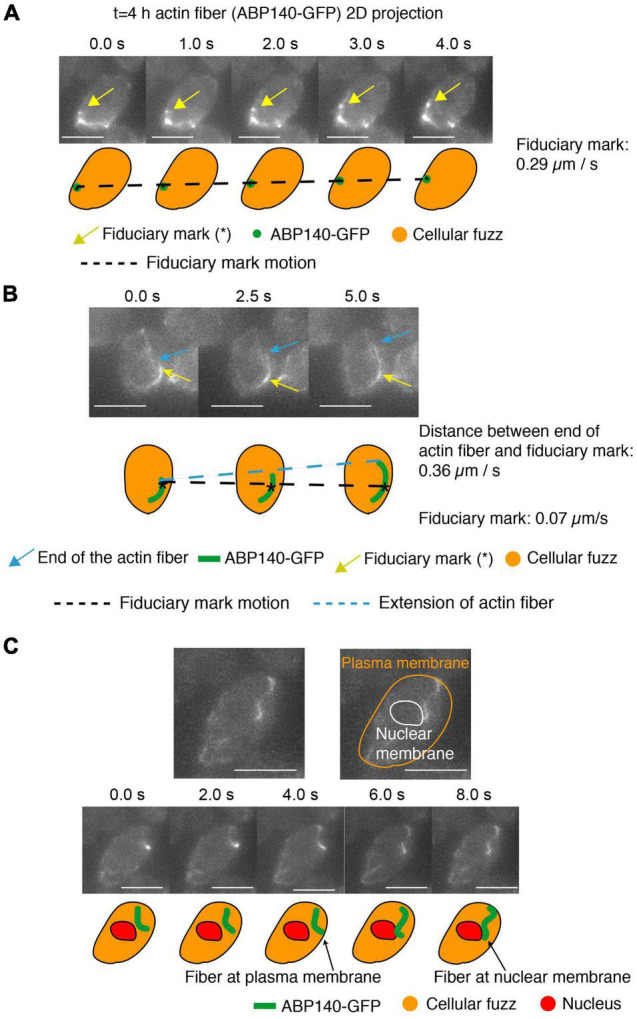
Actin fibers in meiotic prophase undergo movement and treadmilling at meiotic prophase (*t* = 4 h). **(A–C)** 2D projected images of actin fibers labeled by ABP140-GFP (green) plus the entire cellular region as defined by non-specifically bound GFP signal (orange). In **(A,B)**, a local intensity comprising a fiduciary mark is indicated by a gold arrow. In **(B)**, one end of an elongating actin fiber is indicated by a blue arrow. In **(C)**, the periphery of the nucleus, and thus the nuclear membrane, is defined by absence of cellular “fuzz.” Cartoons of these features are shown below. In **(A)**, the fiduciary mark moves 1.16 μm during the interval from 0 to 4 s (0.29 μm/s). In **(B)** The length between the fiduciary mark and the end of the actin fiber extends 1.82 μm from 0 to 5 s (0.36 μm/s) and the fiduciary mark is essentially immobile (position change of 0.07 μm/s). **(C)** The actin fiber movement from the plasma membrane to the nuclear envelope, which is the dark region (red). Scale bar is 5 μm.

We observed movement of a fiduciary mark, and thus the actin fiber, at ∼0.29 μm per sec and 0.26 μm per sec (Figure 4A and Supplementary Figure 1A). This is close to the 0.52 μm/s movement reported in mitotic cells (Yang and Pon, 2002). Movements in mitotic/meiotic cells include cases in which the actin fibers are associated either along the outer nuclear periphery, presumptively by association with the outer nuclear membrane (Koszul et al., 2008; Supplementary Figure 1C), or along the cell periphery, in accord with the well-known association of actin with the plasma membrane (Moseley and Goode, 2006). These dual localizations are illustrated here for meiotic prophase by an example in which an actin fiber moves from association with the edge of the cell (defined by cellular “fuzz”) to the outer surface of the nucleus (defined by the absence of cellular “fuzz”) (Figure 4C).

Finally, we can also observe treadmilling. Here, the distance between a fiduciary mark and the one end of a filament increased at ∼0.36 μm per sec and ∼0.15 μm per sec (Figure 4B and Supplementary Figure 1A), similarly to the ∼0.3 μm/s rate defined for this process in mitotic cells (Yang and Pon, 2002). We note, however, that (contrary to previous considerations; Koszul et al., 2008; Lee et al., 2020), treadmilling cannot contribute to telomere movement, because addition/subtraction at the ends of a filament will not affect the position of a centrally positioned telomere/LINC/Myo2/actin complex.

## Discussion

Dynamic movements of chromosomes at mid-prophase of meiosis are the result of cytoskeleton-mediate motions of chromosome ends, an effect that requires transduction of forces through the nuclear envelope. Here, 3D low SNR imaging in budding yeast has allowed visualization of meiotic telomere movement over extended time periods at high resolution in time and space. Analysis of telomere displacements vs. time plus spatio-temporal analysis of trajectories define three categories of motion which can be directly related to telomere behaviors manifested by per-nucleus whole chromosome motions as described previously: directly promoted Myo2/actin-mediated movement; pauses in which the telomere “jiggles in place”; and periods when the monitored telomere is being moved due to indirect effects resulting from directly promoted movement of an unmonitored telomere. Detailed consideration of these three types of movement raises several interesting issues. In addition, these studies are enabled by a new method for low SNR imaging which has broad potential for further elucidation of chromosome dynamics in yeast and other systems.

### Myo2-Mediated Active Transport

A marked telomere sporadically undergoes fast, brief, straight/curvilinear motion. Myosin protein Myo2, which translocates along actin fibers, has been implicated as a major mediator of prophase movements (Lee et al., 2020). The movements defined in the present study as Group 1 by step-size analysis and as “straight” motion in phenotypic analysis, match those attributed to direct, active Myo2/actin-mediated transport in other systems. We observe average speeds of telomere movement of ∼0.5 μm per 500 ms step, with speeds as high as ∼1.3 μm per 500 ms step observed in some cases (Figures 2E,J). The speed of Myo2-mediated motion along a filament varies with its cargo, but has been reported to be as high as ∼1–3 μm/s in mitotic cells (Beach et al., 2000; Schott et al., 2002).

We also demonstrate that actin fiber localization is as dynamic during meiotic prophase as previously reported for mitotic cells. This is important because the speed of a particular Myo2-mediated event will reflect the combined rates of directional Myo2 tracking along an actin fiber, from the + end to the − end of the filament (Förtsch et al., 2011), and of movement of the fiber itself. Thus, actin fiber movement may either add to, or subtract from, the Myo2 tracking rate. This interplay is likely an important contributor to the range of rates of motion observed for very fast (directly promoted) telomere movements.

We also note that myosins are capable of smoothly negotiating actin fiber branches or switching smoothly from one fiber to another, without apparent disruption of movement (Hammer and Wu, 2007). Such effects could explain cases in which two straight trajectories are linked by an abrupt turn (e.g., Figure 2M, single asterisk).

### What Are Telomeres Doing When They Are Not Engaged in Active Transport?

The current analysis provides further evidence that Myo2/actin-mediated motion is, for a given telomere, quite sporadic. Step size analysis suggests that these motions occupy only ∼17% of the telomere’s life (Figure 2H). This finding matches previous evidence from whole chromosome analysis which also suggests that, at any given moment, among the ∼32 telomeres within a given mid-prophase nucleus, only one or two are undergoing actin-mediated motion (Koszul et al., 2008; Supplementary Figure 1B). This feature, which is likely important for the *in vivo* roles of motion, is not unexpected given that actin fibers only contact the nuclear periphery in a few places and transiently.

These considerations also emphasize the important fact that a given individual telomere spends most of its time in a “resting state” where it may be either motionless (“paused”) or undergoing passive motion promoted indirectly by direct actin-mediated motion of some other (nearby) telomere (“indirect movement”). In the current study, the marked telomere spends ∼85% of its time in one or the other of these states. Moreover, telomeres exhibit the same rates of movement (as defined by 500 ms step sizes) in both conditions. This correspondence suggests, as might be expected *a priori*, that the only difference between these two states is whether the monitored telomere is, or is not, subjected to indirect effects from active movement of some other telomeres.

Two further, related questions thus arise:

(1) What is the state of the (many) telomeres that are not undergoing direct, actin-mediated movement? The presented findings show that these telomeres exhibit 500 ms step sizes which are similar or identical to those observed in an *ndj1Δ* mutant. Since telomeres are not associated with LINC complexes in this mutant, the simplest hypothesis is that the same is true for telomeres in the “resting state.”
(2) How do the molecular ensembles that undergo Myo2/actin-mediated motion manage to assemble? Several lines of evidence point to concerted assembly of telomere/Ndj1– LINC—Myo2—actin complexes (Conrad et al., 2007, 2008; Scherthan et al., 2007; Kosaka et al., 2008; Wanat et al., 2008; Lee et al., 2012, 2020; Fan et al., 2020). Furthermore, it would be sensible if such complexes only assembled when the possibility of actin-mediated motion exists. Thus, an interesting possibility could be that assembly of the necessary complexes, including capture of telomeres by a LINC ensemble, is triggered by direct contact between an actin filament and the nuclear envelope.

### Significance and Basis of Telomere Transport-Mediated Nuclear Shape Deformations

Simultaneous visualization of telomere position and nuclear shape has provided new information regarding the interplay of actin-mediated telomere movement and nuclear shape. We can document cases in which Myo2/actin-mediated movement of the monitored telomere is correlated with, and apparently responsible for, global nuclear envelope deformation. The same relationship is prominent in whole chromosome analysis where telomere-led motion results in indirect movement of multiple nearby chromosomes, thereby changing the entire shape of the chromosome complement and thus, presumptively, the nuclear envelope (Supplementary Figure 1B). The phenomenon of global nuclear envelope deformation raises two important questions.

(1) Directly promoted telomere movements are always very transient. What limits the duration of these events? Myo2-transported LINC/telomere complex will necessarily drag with it the associated nuclear envelope segment. Thus, the extent/duration of active transport might be limited by resistance from associated nuclear envelope deformation. The yeast nuclear envelope should be sufficiently coherent to resist Myo2-mediated pulling forces despite the absence of lamins (Meseroll and Cohen-Fix, 2016; Agrawal and Lele, 2020).
(2) What is the basis for global deformations of nuclear shape? Nuclear shape changes are correlated with movements of multiple chromosomes that occur as an indirect consequence of telomere-led actin-mediated movements (above). We further show here that, after telomere-led nucleus elongation, release of that telomere from the actin fiber results in rapid recoil of the telomere and an associated return of the nuclear envelope to a less-deformed state (Figure 3H and Supplementary Figure 1B). We previously proposed that coordinate motion results from intra-chromosomal linkages among the involved chromosomes (Koszul et al., 2008). By this hypothesis, nuclear envelope shape changes would result indirectly from changes in chromosome relationships. However, we can now suggest the alternative possibility that telomere-led nuclear envelope deformation results in correlated movement of other non-actin-associated telomeres and thereby promotes their coordinate motions without involvement of direct inter-chromosomal connectedness. Another interesting phenomenon revealed by the motion phenotype analysis

is that the trajectories of indirectly promoted movement last longer (5–15 s) than those of the directly promoted motion (∼3 s) by which they are provoked. This distinction could be explained by the viscoelasticity of the secondary motions, which can be expected from the properties of either (both) the nuclear envelope and/or inter-chromosomal connections.

### Low Signal-to-Noise Ratio Imaging Methodology

The methodology used in the current study depends on specialized imaging methods and a novel denoising algorithm in which a two-stage maximum likelihood pipeline is used to accurately detect very faint spots (Kleckner and Chang, 2017; Chang, 2018). These approaches have significant advantages with respect to those described previously for both short time-scale and long time-scale imaging. In the present study we mainly exploited short this method for spot detection for rapid imaging over time scales of minutes (∼500 ms per single 3D stack for ∼6 min). However, it is now technically possible to carry out long time-scale 3D imaging from pre-meiotic G1/G0 to the MI/MII divisions and beyond, over a period of up to 10 h or more, with 3D images taken at 1 min intervals and with low photobleaching and low phototoxicity (Supplementary Figures 4A–C). In addition, two-color 3D imaging is also possible, both for short and long-time scales. Our methods should thus provide a powerful tool to further investigate many processes of interest, including (but not limited to) meiotic pairing, chromosome structure, chromosome movement, and nuclear envelope mechanics and dynamics.

## Data Availability Statement

The raw data supporting the conclusions of this article will be made available by the authors, without undue reservation.

## Author Contributions

TN and BW constructed the strains. TN performed imaging and image analysis. FC built the imaging system and wrote the computer algorithm. TN and NK wrote the article. All authors contributed to the article and approved the submitted version.

## Conflict of Interest

The authors declare that the research was conducted in the absence of any commercial or financial relationships that could be construed as a potential conflict of interest.

## Publisher’s Note

All claims expressed in this article are solely those of the authors and do not necessarily represent those of their affiliated organizations, or those of the publisher, the editors and the reviewers. Any product that may be evaluated in this article, or claim that may be made by its manufacturer, is not guaranteed or endorsed by the publisher.

## References

[B1] Agrawal A. Lele T. P. (2020). Geometry of the nuclear envelope determines its flexural stiffness. *Mol. Biol. Cell* 31 1815–1821. 10.1091/mbc.E20-02-0163 32583742 PMC7521844

[B2] Baudrimont A. Penkner A. Woglar A. Machacek T. Wegrostek C. Gloggnitzer J. (2010). Leptotene/zygotene chromosome movement via the SUN/KASH protein bridge in *Caenorhabditis elegans*. *PLoS Genet.* 6:e1001219. 10.1371/journal.pgen.1001219 21124819 PMC2991264

[B3] Beach D. L. Thibodeaux J. Maddox P. Yeh E. Bloom K. (2000). The role of the proteins Kar9 and Myo2 in orienting the mitotic spindle of budding yeast. *Curr. Biol.* 10 1497–1506. 11114516 10.1016/s0960-9822(00)00837-x

[B4] Benaglia T. Chauveau D. Hunter D. R. Young D. S. (2009). mixtools: an R package for analyzing mixture models. *J. Stat. Softw.* 32 1–29. 10.4236/oalib.1101815

[B5] Bitran A. Chiang W.-Y. Levine E. Prentiss M. (2017). Mechanisms of fast and stringent search in homologous pairing of double-stranded DNA. *PLoS Comput. Biol.* 13:e1005421. 10.1371/journal.pcbi.1005421 28257444 PMC5360337

[B6] Chang F. (2018). *Low SNR Computational Pattern Detection Applied to Multi-Spectral 3D Molecular Dynamics.* Available online at: https://dash.harvard.edu/handle/1/42015127 (accessed March 28, 2021).

[B7] Chen J. Zhang Z. Li L. Chen B.-C. Revyakin A. Hajj B. (2014). Single-molecule dynamics of enhanceosome assembly in embryonic stem cells. *Cell* 156 1274–1285. 10.1016/j.cell.2014.01.062 24630727 PMC4040518

[B8] Chikashige Y. Ding D. Q. Funabiki H. Haraguchi T. Mashiko S. Yanagida M. (1994). Telomere-led premeiotic chromosome movement in fission yeast. *Science* 264 270–273. 10.1126/science.8146661 8146661

[B9] Chikashige Y. Tsutsumi C. Yamane M. Okamasa K. Haraguchi T. Hiraoka Y. (2006). Meiotic proteins bqt1 and bqt2 tether telomeres to form the bouquet arrangement of chromosomes. *Cell* 125 59–69. 10.1016/j.cell.2006.01.048 16615890

[B10] Christophorou N. Rubin T. Bonnet I. Piolot T. Arnaud M. Huynh J.-R. (2015). Microtubule-driven nuclear rotations promote meiotic chromosome dynamics. *Nat. Cell Biol.* 17 1388–1400. 10.1038/ncb3249 26458247

[B11] Chubb J. R. Boyle S. Perry P. Bickmore W. A. (2002). Chromatin motion is constrained by association with nuclear compartments in human cells. *Curr. Biol.* 12 439–445. 10.1016/s0960-9822(02)00695-4 11909528

[B12] Conrad M. N. Lee C.-Y. Chao G. Shinohara M. Kosaka H. Shinohara A. (2008). Rapid telomere movement in meiotic prophase is promoted by NDJ1, MPS3, and CSM4 and is modulated by recombination. *Cell* 133 1175–1187. 10.1016/j.cell.2008.04.047 18585352

[B13] Conrad M. N. Lee C.-Y. Wilkerson J. L. Dresser M. E. (2007). MPS3 mediates meiotic bouquet formation in Saccharomyces cerevisiae. *Proc. Natl. Acad. Sci. U.S.A.* 104 8863–8868. 10.1073/pnas.0606165104 17495028 PMC1885593

[B14] Davis L. Smith G. R. (2006). The meiotic bouquet promotes homolog interactions and restricts ectopic recombination in *Schizosaccharomyces pombe*. *Genetics* 174 167–177. 10.1534/genetics.106.059733 16988108 PMC1569800

[B15] Dion V. Kalck V. Horigome C. Towbin B. D. Gasser S. M. (2012). Increased mobility of double-strand breaks requires Mec1, Rad9 and the homologous recombination machinery. *Nat. Cell Biol.* 14 502–509. 10.1038/ncb2465 22484486

[B16] Fan J. Jin H. Koch B. A. Yu H.-G. (2020). Mps2 links Csm4 and Mps3 to form a telomere-associated LINC complex in budding yeast. *Life Sci Alliance* 3:e202000824. 10.26508/lsa.202000824 32967926 PMC7536833

[B17] Förtsch J. Hummel E. Krist M. Westermann B. (2011). The myosin-related motor protein Myo2 is an essential mediator of bud-directed mitochondrial movement in yeast. *J. Cell Biol.* 194 473–488. 10.1083/jcb.201012088 21807878 PMC3153652

[B18] Hajjoul H. Mathon J. Ranchon H. Goiffon I. Mozziconacci J. Albert B. (2013). High-throughput chromatin motion tracking in living yeast reveals the flexibility of the fiber throughout the genome. *Genome Res.* 23 1829–1838. 10.1101/gr.157008.113 24077391 PMC3814883

[B19] Hammer J. A. III Wu X. (2007). Slip sliding away with myosin V. *Proc. Natl. Acad. Sci. U.S.A.* 104 5255–5256. 10.1073/pnas.0701071104 17374717 PMC1838462

[B20] Heun P. Laroche T. Shimada K. Furrer P. Gasser S. M. (2001). Chromosome dynamics in the yeast interphase nucleus. *Science* 294 2181–2186. 10.1126/science.1065366 11739961

[B21] Izeddin I. Récamier V. Bosanac L. Cissé I. I. Boudarene L. Dugast-Darzacq C. (2014). Single-molecule tracking in live cells reveals distinct target-search strategies of transcription factors in the nucleus. *Elife* 3:e02230. 10.7554/eLife.02230 24925319 PMC4095940

[B22] Kim K. P. Weiner B. M. Zhang L. Jordan A. Dekker J. Kleckner N. (2010). Sister cohesion and structural axis components mediate homolog bias of meiotic recombination. *Cell* 143 924–937. 10.1016/j.cell.2010.11.015 21145459 PMC3033573

[B23] Kleckner N. Weiner B. M. (1993). Potential advantages of unstable interactions for pairing of chromosomes in meiotic, somatic, and premeiotic cells. *Cold Spring Harb. Symp. Quant. Biol.* 58 553–565. 10.1101/sqb.1993.058.01.062 7956070

[B24] Kleckner N. E. Chang F. S. (2017). *Pattern Detection at Low Signal-to-Noise Ratio. World Patent.* Available online at: https://patentimages.storage.googleapis.com/43/62/72/6fd46ce0c45b38/WO2017040669A1.pdf (accessed September 27, 2021).

[B25] Kosaka H. Shinohara M. Shinohara A. (2008). Csm4-dependent telomere movement on nuclear envelope promotes meiotic recombination. *PLoS Genet.* 4:e1000196. 10.1371/journal.pgen.1000196 18818742 PMC2533704

[B26] Koszul R. Kim K. P. Prentiss M. Kleckner N. Kameoka S. (2008). Meiotic chromosomes move by linkage to dynamic actin cables with transduction of force through the nuclear envelope. *Cell* 133 1188–1201. 10.1016/j.cell.2008.04.050 18585353 PMC2601696

[B27] Koszul R. Kleckner N. (2009). Dynamic chromosome movements during meiosis: a way to eliminate unwanted connections? *Trends Cell Biol.* 19 716–724. 10.1016/j.tcb.2009.09.007 19854056 PMC2787882

[B28] Lee C.-Y. Bisig C. G. Conrad M. M. Ditamo Y. Previato de Almeida L. Dresser M. E. (2020). Extranuclear structural components that mediate dynamic chromosome movements in yeast meiosis. *Curr. Biol.* 30 1207–1216.e4. 10.1016/j.cub.2020.01.054 32059771 PMC7181386

[B29] Lee C.-Y. Conrad M. N. Dresser M. E. (2012). Meiotic chromosome pairing is promoted by telomere-led chromosome movements independent of bouquet formation. *PLoS Genet.* 8:e1002730. 10.1371/journal.pgen.1002730 22654677 PMC3359977

[B30] Lee C.-Y. Horn H. F. Stewart C. L. Burke B. Bolcun-Filas E. Schimenti J. C. (2015). Mechanism and regulation of rapid telomere prophase movements in mouse meiotic chromosomes. *Cell Rep.* 11 551–563. 10.1016/j.celrep.2015.03.045 25892231 PMC4417006

[B31] Link J. Jantsch V. (2019). Meiotic chromosomes in motion: a perspective from *Mus musculus* and *Caenorhabditis elegans*. *Chromosoma* 128 317–330. 10.1007/s00412-019-00698-5 30877366 PMC6823321

[B32] Link J. Paouneskou D. Velkova M. Daryabeigi A. Laos T. Labella S. (2018). Transient and partial nuclear lamina disruption promotes chromosome movement in early meiotic prophase. *Dev. Cell* 45 212–225.e7. 10.1016/j.devcel.2018.03.018 29689196 PMC5920155

[B33] Marshall W. F. Straight A. Marko J. F. Swedlow J. Dernburg A. Belmont A. (1997). Interphase chromosomes undergo constrained diffusional motion in living cells. *Curr. Biol.* 7 930–939. 10.1016/s0960-9822(06)00412-x 9382846

[B34] Mazza D. Abernathy A. Golob N. Morisaki T. McNally J. G. (2012). A benchmark for chromatin binding measurements in live cells. *Nucleic Acids Res.* 40 e119. 10.1093/nar/gks701 22844090 PMC3424588

[B35] Meseroll R. A. Cohen-Fix O. (2016). The malleable nature of the budding yeast nuclear envelope: flares, fusion, and fenestrations. *J. Cell. Physiol.* 231 2353–2360. 10.1002/jcp.25355 26909870 PMC6260970

[B36] Miné-Hattab J. Recamier V. Izeddin I. Rothstein R. Darzacq X. (2017). Multi-scale tracking reveals scale-dependent chromatin dynamics after DNA damage. *Mol. Biol. Cell* 28 3323–3332. 10.1091/mbc.E17-05-0317 28794266 PMC5687033

[B37] Miné-Hattab J. Rothstein R. (2012). Increased chromosome mobility facilitates homology search during recombination. *Nat. Cell Biol.* 14 510–517. 10.1038/ncb2472 22484485

[B38] Moseley J. B. Goode B. L. (2006). The yeast actin cytoskeleton: from cellular function to biochemical mechanism. *Microbiol. Mol. Biol. Rev.* 70 605–645. 10.1128/mmbr.00013-06 16959963 PMC1594590

[B39] Neumann F. R. Dion V. Gehlen L. R. Tsai-Pflugfelder M. Schmid R. Taddei A. (2012). Targeted INO80 enhances subnuclear chromatin movement and ectopic homologous recombination. *Genes Dev.* 26 369–383. 10.1101/gad.176156.111 22345518 PMC3289885

[B40] Niwa O. Shimanuki M. Miki F. (2000). Telomere-led bouquet formation facilitates homologous chromosome pairing and restricts ectopic interaction in fission yeast meiosis. *EMBO J.* 19 3831–3840. 10.1093/emboj/19.14.3831 10899136 PMC313979

[B41] Normanno D. Boudarène L. Dugast-Darzacq C. Chen J. Richter C. Proux F. (2015). Probing the target search of DNA-binding proteins in mammalian cells using TetR as model searcher. *Nat. Commun.* 6:7357. 10.1038/ncomms8357 26151127 PMC4507003

[B42] Nozaki T. Imai R. Tanbo M. Nagashima R. Tamura S. Tani T. (2017). Dynamic organization of chromatin domains revealed by super-resolution live-cell imaging. *Mol. Cell* 67 282–293.e7. 10.1016/j.molcel.2017.06.018 28712725

[B43] Nozaki T. Kaizu K. Pack C.-G. Tamura S. Tani T. Hihara S. (2013). Flexible and dynamic nucleosome fiber in living mammalian cells. *Nucleus* 4 349–356. 10.4161/nucl.26053 23945462 PMC3899123

[B44] Parvinen M. Söderström K. O. (1976). Chromosome rotation and formation of synapsis. *Nature* 260 534–535. 10.1038/260534a0 1264213

[B45] Penkner A. Tang L. Novatchkova M. Ladurner M. Fridkin A. Gruenbaum Y. (2007). The nuclear envelope protein Matefin/SUN-1 is required for homologous pairing in *C. elegans* meiosis. *Dev. Cell* 12 873–885. 10.1016/j.devcel.2007.05.004 17543861

[B46] Robinett C. C. Straight A. Li G. Willhelm C. Sudlow G. Murray A. (1996). In vivo localization of DNA sequences and visualization of large-scale chromatin organization using lac operator/repressor recognition. *J. Cell Biol.* 135 1685–1700. 8991083 10.1083/jcb.135.6.1685 PMC2133976

[B47] Sage D. Neumann F. R. Hediger F. Gasser S. M. Unser M. (2005). Automatic tracking of individual fluorescence particles: application to the study of chromosome dynamics. *IEEE Trans. Image Process.* 14 1372–1383. 10.1109/tip.2005.852787 16190472

[B48] Sato A. Isaac B. Phillips C. M. Rillo R. Carlton P. M. Wynne D. J. (2009). Cytoskeletal forces span the nuclear envelope to coordinate meiotic chromosome pairing and synapsis. *Cell* 139 907–919. 10.1016/j.cell.2009.10.039 19913287 PMC2825574

[B49] Scherthan H. Wang H. Adelfalk C. White E. J. Cowan C. Cande W. Z. (2007). Chromosome mobility during meiotic prophase in *Saccharomyces cerevisiae*. *Proc. Natl. Acad. Sci. U.S.A.* 104 16934–16939. 10.1073/pnas.0704860104 17939997 PMC2040470

[B50] Schindelin J. Arganda-Carreras I. Frise E. Kaynig V. Longair M. Pietzsch T. (2012). Fiji: an open-source platform for biological-image analysis. *Nat. Methods* 9 676–682. 10.1038/nmeth.2019 22743772 PMC3855844

[B51] Schott D. H. Collins R. N. Bretscher A. (2002). Secretory vesicle transport velocity in living cells depends on the myosin-V lever arm length. *J. Cell Biol.* 156 35–39. 10.1083/jcb.200110086 11781333 PMC2173574

[B52] Seeber A. Dion V. Gasser S. M. (2013). Checkpoint kinases and the INO80 nucleosome remodeling complex enhance global chromatin mobility in response to DNA damage. *Genes Dev.* 27 1999–2008. 10.1101/gad.222992.113 24029917 PMC3792476

[B53] Sheehan M. J. Pawlowski W. P. (2009). Live imaging of rapid chromosome movements in meiotic prophase I in maize. *Proc. Natl. Acad. Sci. U.S.A.* 106 20989–20994. 10.1073/pnas.0906498106 19926853 PMC2791616

[B54] Shibuya H. Ishiguro K.-I. Watanabe Y. (2014). The TRF1-binding protein TERB1 promotes chromosome movement and telomere rigidity in meiosis. *Nat. Cell Biol.* 16 145–156. 10.1038/ncb2896 24413433

[B55] Slutsky M. Mirny L. A. (2004). Kinetics of protein-DNA interaction: facilitated target location in sequence-dependent potential. *Biophys. J.* 87 4021–4035. 10.1529/biophysj.104.050765 15465864 PMC1304911

[B56] Storlazzi A. Gargano S. Ruprich-Robert G. Falque M. David M. Kleckner N. (2010). Recombination proteins mediate meiotic spatial chromosome organization and pairing. *Cell* 141 94–106. 20371348 10.1016/j.cell.2010.02.041 PMC2851631

[B57] Straight A. F. Belmont A. S. Robinett C. C. Murray A. W. (1996). GFP tagging of budding yeast chromosomes reveals that protein–protein interactions can mediate sister chromatid cohesion. *Curr. Biol.* 6 1599–1608. 10.1016/s0960-9822(02)70783-5 8994824

[B58] Tinevez J.-Y. Perry N. Schindelin J. Hoopes G. M. Reynolds G. D. Laplantine E. (2017). TrackMate: an open and extensible platform for single-particle tracking. *Methods* 115 80–90. 10.1016/j.ymeth.2016.09.016 27713081

[B59] Vazquez J. Belmont A. S. Sedat J. W. (2001). Multiple regimes of constrained chromosome motion are regulated in the interphase *Drosophila nucleus*. *Curr. Biol.* 11 1227–1239. 10.1016/s0960-9822(01)00390-6 11525737

[B60] Wanat J. J. Kim K. P. Koszul R. Zanders S. Weiner B. Kleckner N. (2008). Csm4, in collaboration with Ndj1, mediates telomere-led chromosome dynamics and recombination during yeast meiosis. *PLoS Genet.* 4:e1000188. 10.1371/journal.pgen.1000188 18818741 PMC2533701

[B61] Wettstein D. Rasmussen S. W. Holm P. B. (1984). The synaptonemal complex in genetic segregation. *Annu. Rev. Genet.* 18 331–411. 10.1146/annurev.ge.18.120184.001555 6241453

[B62] Wynne D. J. Rog O. Carlton P. M. Dernburg A. F. (2012). Dynein-dependent processive chromosome motions promote homologous pairing in *C. elegans* meiosis. *J. Cell Biol.* 196 47–64. 10.1083/jcb.201106022 22232701 PMC3255982

[B63] Yancey-Wrona J. E. Camerini-Otero R. D. (1995). The search for DNA homology does not limit stable homologous pairing promoted by RecA protein. *Curr. Biol.* 5 1149–1158. 10.1016/s0960-9822(95)00231-4 8548287

[B64] Yang H.-C. Pon L. A. (2002). Actin cable dynamics in budding yeast. *Proc. Natl. Acad. Sci. U.S.A.* 99 751–756. 10.1073/pnas.022462899 11805329 PMC117377

[B65] Zickler D. (2006). From early homologue recognition to synaptonemal complex formation. *Chromosoma* 115 158–174. 10.1007/s00412-006-0048-6 16570189

[B66] Zickler D. Kleckner N. (1999). Meiotic chromosomes: integrating structure and function. *Annu. Rev. Genet.* 33 603–754. 10.1146/annurev.genet.33.1.603 10690419

[B67] Zickler D. Kleckner N. (2015). Recombination, pairing, and synapsis of homologs during meiosis. *Cold Spring Harb. Perspect. Biol.* 7:a016626. 10.1101/cshperspect.a016626 25986558 PMC4448610

