# Supplementary material for: High Temporal Resolution 3D Live-Cell Imaging of Budding Yeast Meiosis Defines Discontinuous Actin/Telomere-Mediated Chromosome Motion, Correlated Nuclear Envelope Deformation and Actin Filament Dynamics
Source: Front Cell Dev Biol. 2021 Nov 25;9:687132. doi: 10.3389/fcell.2021.687132 (PMC8656277; doi:10.3389/fcell.2021.687132)
Supplement: Supplementary file 1 [file Data_Sheet_1.pdf]

Supplementary Notes

1. Accuracy of spot detection and localization.

To evaluate the localization accuracy in this system, including both drift of the imaging sample and accuracy of the spot detection, we observed the tagged *SCP1* locus in paraformaldehyde (PFA)-fixed cells (Supplementary Figure 2A; n = 2 cells; Nozaki et al., 2013; Miné-Hattab et al., 2017). Movement of the tagged *SCP1* locus in a fixed cell is very limited as compared to that observed even at G1/G0 as seen by a representative 3D trace (Supplementary Figure 2A) and the plateau of the MSD curve implies strong confinement, with Rc = 0.14 μm (Supplementary Figure 2B, compared with 0.64 μm and 1.47 μm at t=0 h and t=4h cells in Figures 1G and 1H). Only in the presence of LatB, where actin is absent and there is essentially no source of active motion, does the Rc even begin to approach that observed in fixed cells (Rc = 0.42 μm). Furthermore, the standard deviation (SD) of the spot position defined in fixed cells over 3 minutes of imaging is: SD_x:0.043 μm, SD_y: 0.035 μm, and SD_z: 0.074 μm. These values, which we estimate to be the upper bound of the localization accuracy, are lower than the average 500ms step sizes observed in either G1/G0 or mid-prophase cells (~0.2 μm; below). Taken together, these control findings confirm that all the imaging results presented reflect the actual motion of the tagged telomere locus.

2. Absence of fluorescent spot bleaching during imaging.

The kymographs of 500 ms 3D imaging over 3min subsets of 6min total imaging times (e.g., Figure 1) demonstrate visually that no significant photobleaching occurs during these time series. However, we have further documented the absence of any significant photobleaching effect on our analysis by a specifically-designed control experiment. We sorted the data of 500 ms telomere spot step sizes into two groups according to whether the images were collected early in the imaging series (0-3min) or later in the series (3-6min). We did this for both G1/G0 data and prophase (t=4h data) from time series analyzed in Figures 2E and Supplementary Figure 3C. The results are indistinguishable in the two cases. Step sizes per 500 ms for cells at t=0: 0-3min: 0.19 μm; 3-6 min: 0.20 μm; total: 0.19 μm. Step sizes per 500 ms

for cells at t=4h: 0-3min: 0.20 μm; 3-6min: 0.20 μm; total: 0.20 μm. These data show that photobleaching (and phototoxicity) is negligible in our imaging conditions and thus do not influence our results.

Supplementary Figure 1.

Further analysis of previously-described motions.

(A) Telomere of pachytene chromosome stained by DAPI (yellow arrowhead) moving along nucleus-hugging actin fiber (ABP140-GFP, green) with fiduciary mark (turquoise arrowhead) (Figure 4E of Koszul et al., 2008). (B) Coordinated chromosome motion at pachytene, presented previously as Figure 2A of Koszul et al., 2008, can now be related to the two types of telomere motions described above. (C) Actin filaments hug the nucleus but extend beyond into the cytoplasm. Three-dimensional reconstruction of actin cables disposition along the nuclei; planes containing the bottom of the nucleus and of the cell are indicated by white arrowhead and dotted square, respectively. (Figure 4B of (Koszul et al., 2008)).
All figures reproduced from (Koszul et al., 2008).

Supplementary Figure 2

(A) Representative trajectories of spots detected and tracked in 3D in fixed cells. (B) MSD curves of the tagged *SCP1* locus in the fixed cells (green), in living cells at pre-meiotic G1/G0 (blue) and meiotic prophase (red) (n = 2 cells for fixed cells, n = 10 cells for t = 0 h and n = 17 cells for t = 4 h). (C) MSD curves with error bar (standard error) of *SCP1* locus in the cells at pre-meiotic G1/G0 (blue) and meiotic prophase (red) (n = 10 cells for t = 0 h and n = 17 cells for t = 4 h).
(D, E) The distribution of distances traveled by the SPC42 spot over 500 ms (left) and 5s (right) (D); and MSD curves of the SPC42 spot (E).

Supplementary Figure 3

(A) The projection image of the totality of positions observed in the XY plane over the duration of the trajectory in a *ndj1*Δ cell is illustrated. (B) Representative 3D displacement pattern for 3 mins with 500 ms step (turquoise) and 5 s step (orange) in a WT cell at t = 0 h (G1/G0). (C, D) Distribution of distances traveled (step size) over 500 ms (C, mean = 0.19 μm) and 5 s intervals (D, mean = 0.32 μm) plotted with

Gaussian model in WT cells at t = 0h (G1/G0) (n = 10 cells). (E) The projection image of the totality of positions observed in the XY plane over the duration of the trajectory in WT cells at t = 0h (G1/G0) is illustrated. (F) Representative 3D displacement pattern for 3 mins with 500 ms step (turquoise) and 5 s step (orange) in a LatB-treated cell at t = 4h. (G, H) Distribution of distances traveled (step size) over 500 ms (G, mean = 0.13 μm) and 5 s intervals (H, mean = 0.20 μm) plotted with Gaussian model in LatB-treated cells at t = 4h (n = 10 cells). (I) The projection image of the totality of positions observed in the XY plane over the duration of the trajectory in LatB-treated cells is illustrated. The scale bar is 2 μm.

Supplementary Figure 4

(A) Long time scale live-cell imaging of the tagged *SCP1* locus in 3D at 1 min intervals from t=0 hours to t=10 hours after transfer to SPM. (B) Positions of the tagged *SCP1* locus as defined at 1 min intervals. The red arrow indicates the time at which images were collected in the studies presented here (t=4 hours), illustrating the fact that this is in the middle of a period of dramatic telomere movement. (C) Telomere association with the nuclear envelope at t=4 hours of meiosis in the time series presented in Panel (A). Projected images and interpretations as in Figure 3.

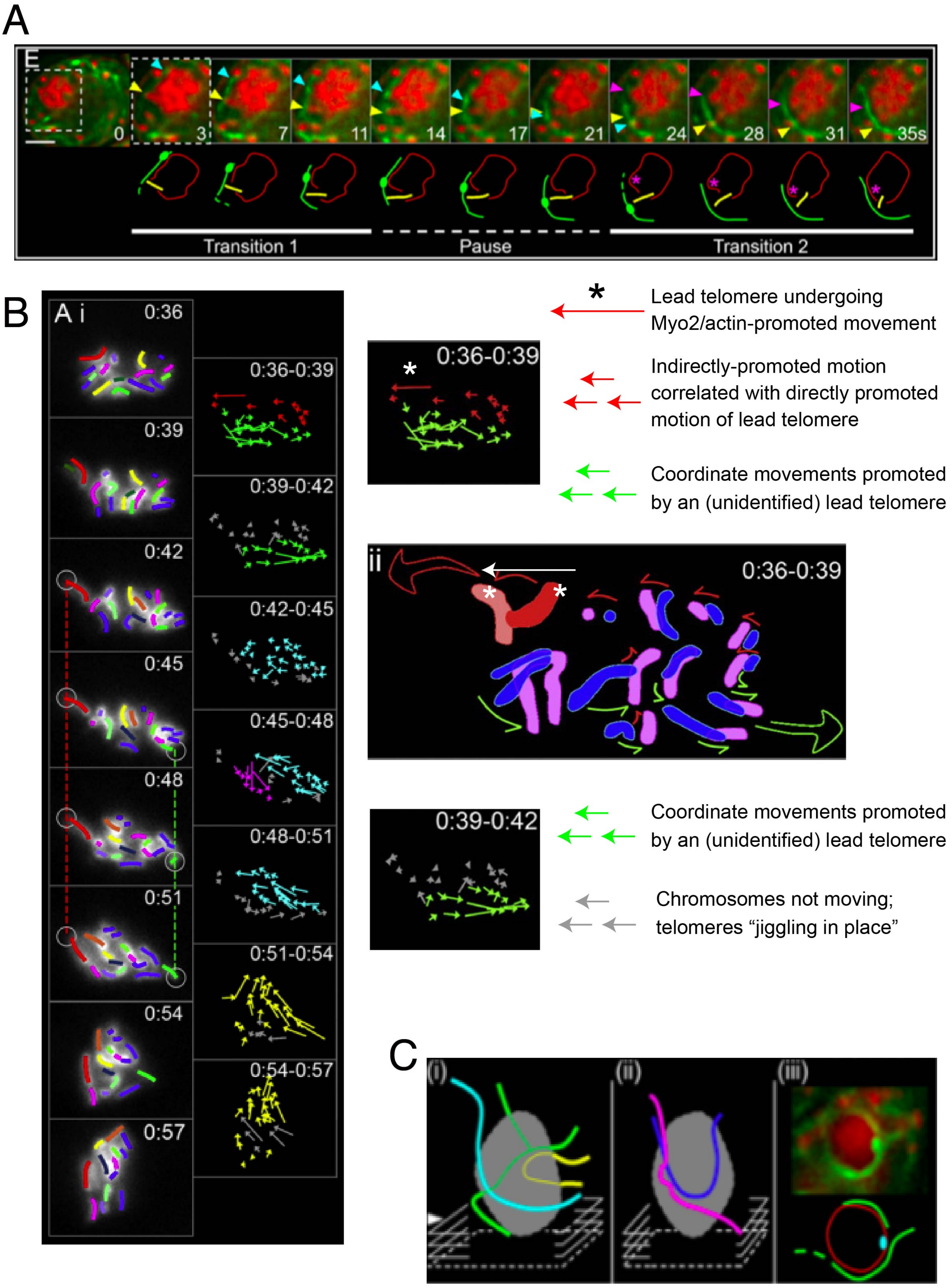

Supplementary Figure 1

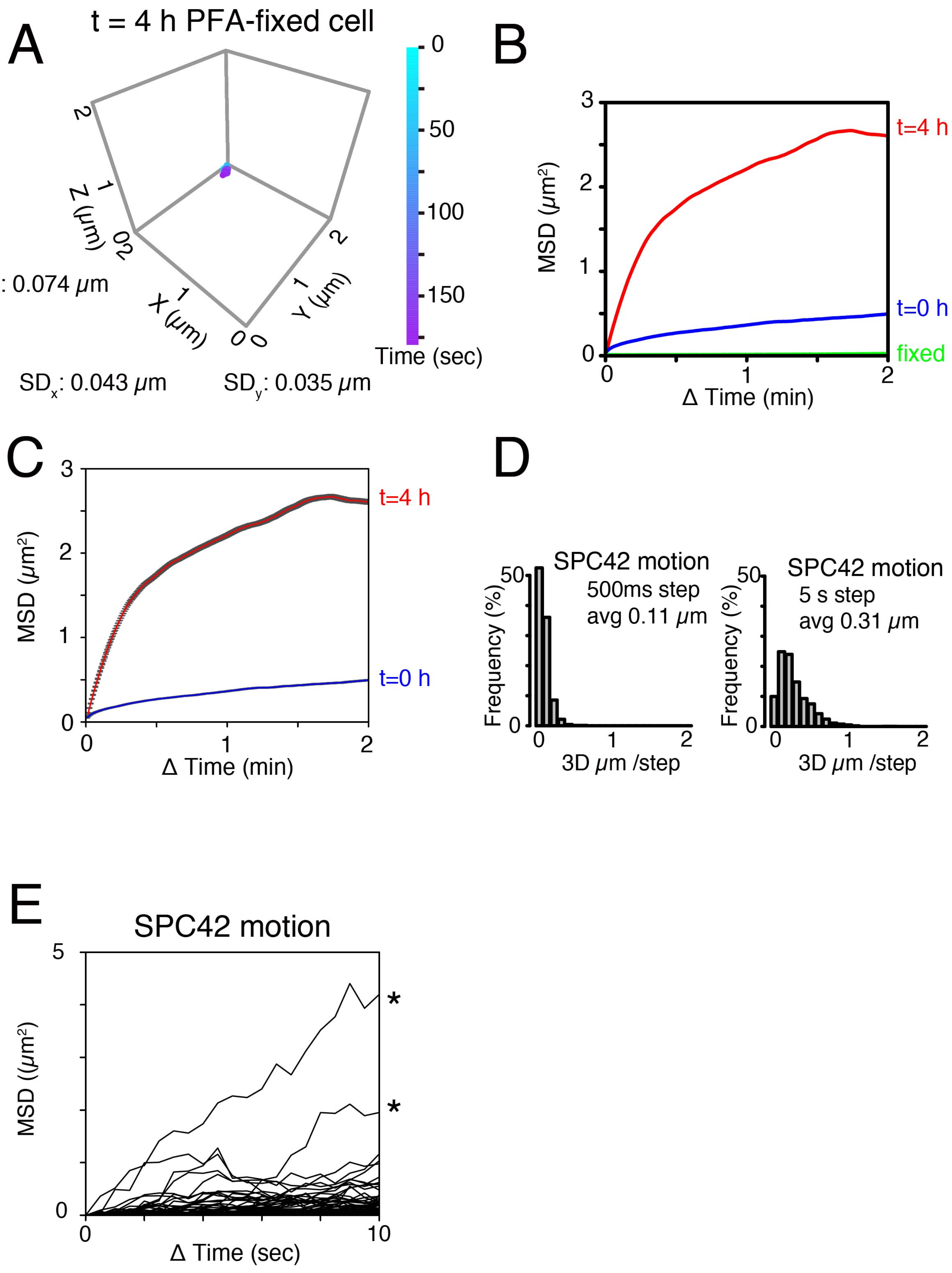

Supplementary Figure 2

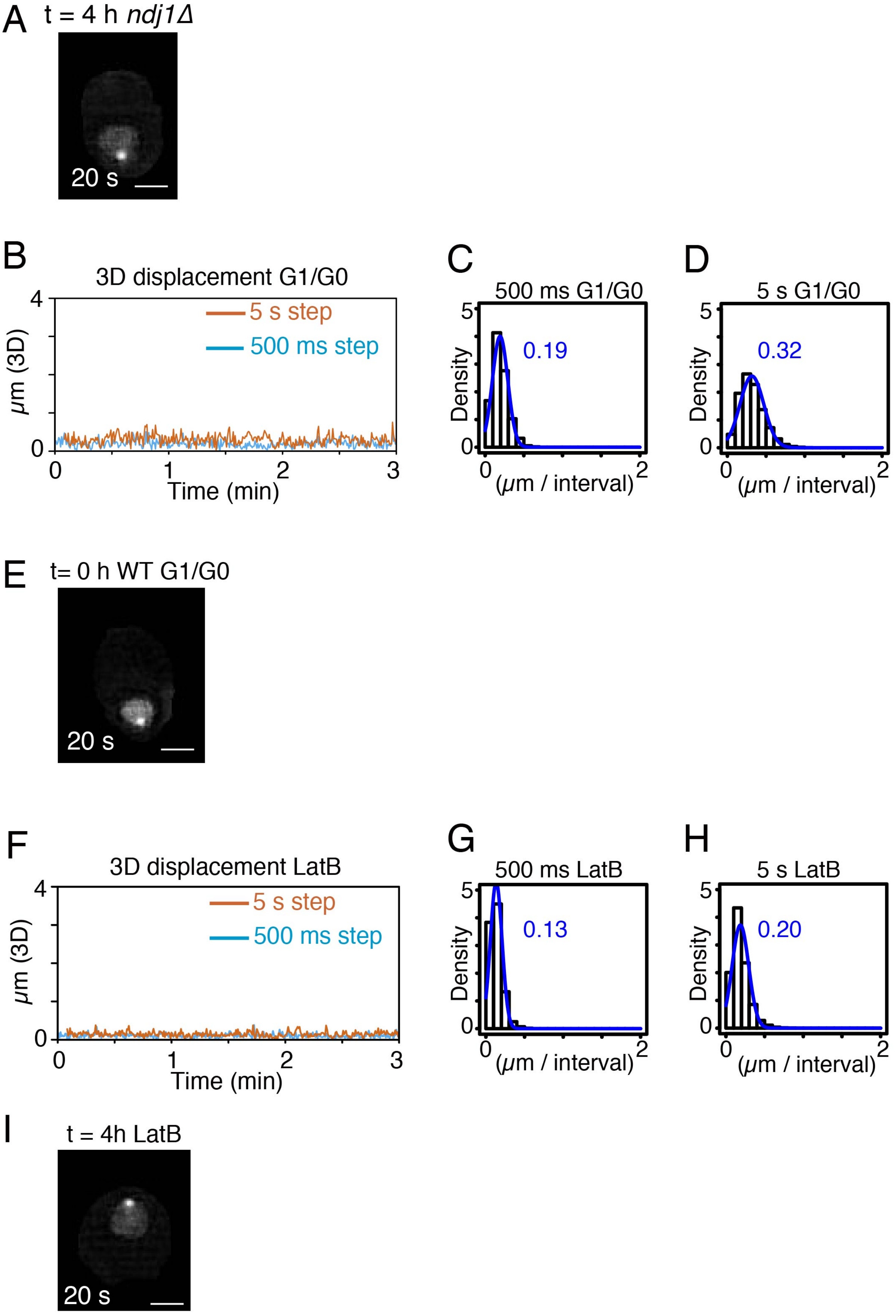

Supplementary Figure 3

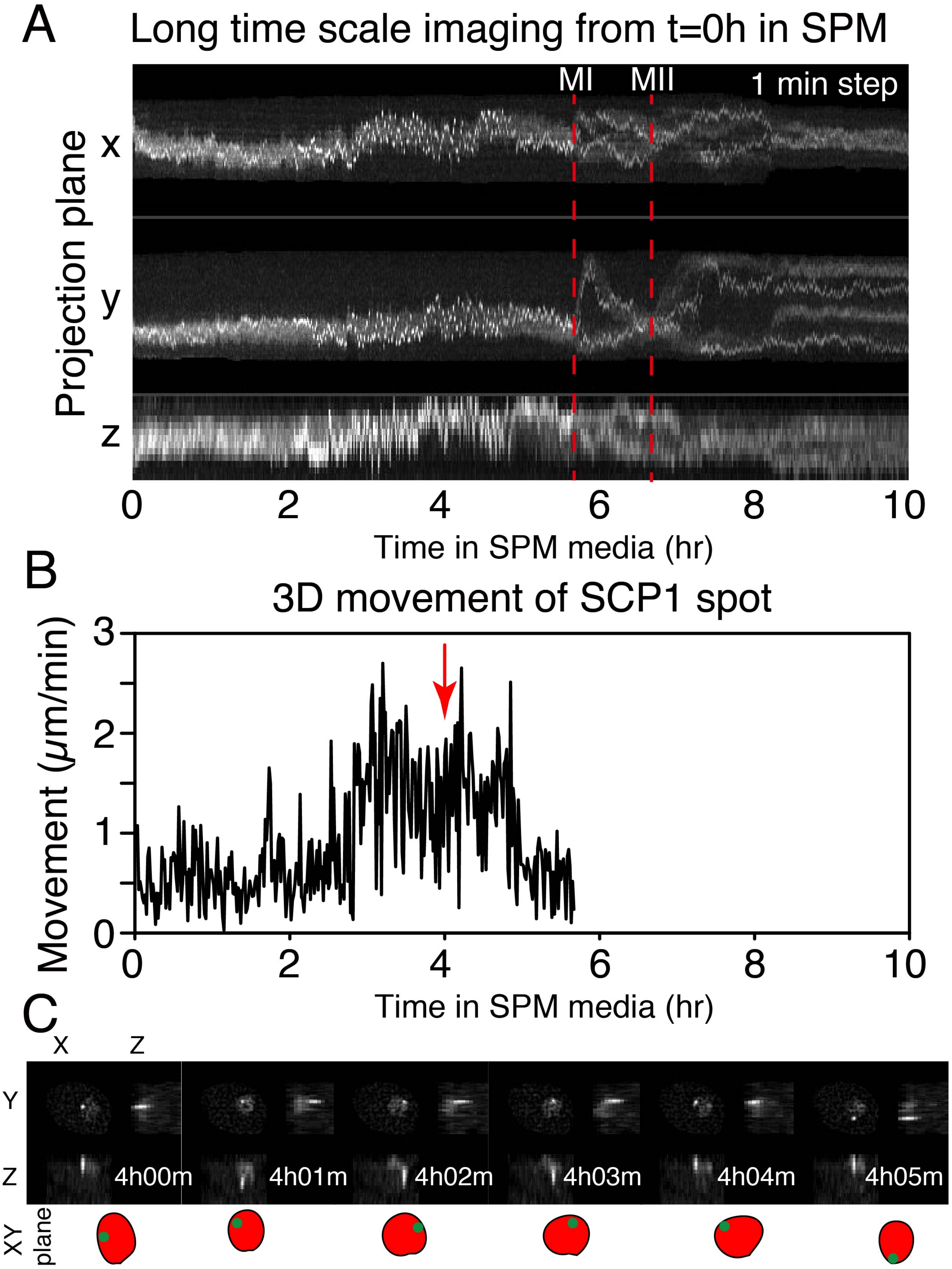

Supplementary Figure 4
